# High glucose-induced cytoplasmic translocation of Dnmt3a contributes to CTGF hypo-methylation in mesangial cells

**DOI:** 10.1042/BSR20160141

**Published:** 2016-08-05

**Authors:** Hao Zhang, Aimei Li, Wei Zhang, Zhijun Huang, Jianwen Wang, Bin Yi

**Affiliations:** * Department of Nephrology, The Third Xiangya Hospital, Central South University, Changsha 410013, Hunan, China

**Keywords:** CTGF, Dnmt, high glucose, human glomerular mesangial cell, methylation

## Abstract

Connective tissue growth factor (CTGF) plays an essential role in the pathogenesis of diabetic nephropathy and we have previously identified that high glucose induced the expression of CTGF by decreasing DNA methylation. The aim of the present study was to investigate the underlying mechanisms of the high glucose-induced CTGF hypo-methylation. Human glomerular mesangial cells (hMSCs) were treated with low glucose (5 mM), mannitol (30 mM) or high glucose (30 mM) respectively. Immunofluorescence staining, real-time quantitative PCR and western blotting were performed to determine the subcellular distribution and expression of CTGF and Dnmt3a. ChIP-PCR assay was applied to investigate the capability of Dnmt3a to bind the CpG island of CTGF. Our results showed that high glucose induced both mRNA and protein expressions of CTGF, and led to increased cytoplasmic translocation of Dnmt3a in cultured hMSCs. The nuclear Dnmt3a protein was significantly reduced after high glucose treatment, although the expression of total Dnmt3a protein was not altered. We further discovered that ERK/MAPK signalling contributed to the high glucose-induced cytoplasmic translocation of Dnmt3a. Consequently, less Dnmt3a protein was bound to the CpG island of CTGF promoter, which induced an increase in CTGF expression by epigenetic regulation in the presence of high glucose. In conclusion, high glucose induces cytoplasmic translocation of Dnmt3a, possibly through activating ERK/MAPK signalling pathway, which contributes to the decreased binding of Dnmt3a on CTGF promoter and the subsequent CTGF hypo-methylation in diabetic nephropathy.

## INTRODUCTION

Diabetic nephropathy (DN) is a major microvascular complication among diabetic patients and the most common cause of end-stage renal disease (ESRD), affecting around 30% of type 1 and type 2 diabetic patients [1]. The major clinical characteristics of DN include progressive proteinuria, renal failure and increased risk of cardiovascular mortality [2]. Prolonged hyperglycaemia plays a crucial role during the diabetic nephropathy development because of its effects in modifying the activities of multiple signalling pathways and transcription factors in mesangial cells and podocytes, which are essential in maintaining the glomerular capillary structure and regulating glomerular filtration. Hyperglycaemia is associated with diffuse thickening of glomerular basement membrane, proliferation and hypertrophy of mesangial cells, podocyte injury, and is a key cause for the loss of glomerular function and the irreversible tubulointerstitial fibrosis [3,4].

Connective tissue growth factor (CTGF), a cysteine-rich protein with molecular weight around 38 kDa, promotes mesangial extracellular matrix synthesis, cellular hypertrophy, cell adhesion and mesangial matrix expansion [5,6]. It is well known as a key factor in the pathogenesis and development of diabetic nephropathy. Up-regulation of CTGF mRNA and protein expressions has been confirmed in high glucose-induced mesangial cells and podocytes [7,8] in both diabetic nephropathy animal models [9,10], and type 1 and type 2 diabetic patients [11,12]. This up-regulation is highly correlated with the severity of albuminuria and the stage of renal insufficiency. It has also been reported that specific blocking of CTGF by antisense oligonucleotide (ASO) significantly decreased proteinuria, reversed mesangial expansion in diabetic mice [13,14] and improved the attenuation of albuminuria in patients with microalbuminuric diabetic kidney disease [14]. These reports further emphasized the importance of CTGF in diabetic nephropathy and demonstrated that inhibition of CTGF might hold substantial promise for the treatment of diabetic nephropathy.

There was emerging evidence indicating that diabetic nephropathy might be extensively regulated by epigenetic modifications [15]. For instance, changes in global histone modifications, which are generally epigenetic markers for active gene transcription, are correlated with progressive glomerulosclerosis in type 2 diabetic mice [16]. Also, CTGF in renal cells is differentially regulated by the inhibition of histone deacetylation [17]. On the other hand, DNA methylation, which takes place on cytosines of CpG island and is catalysed by DNA methyltransferases (Dnmts), usually leads to gene silencing and is also closely related to diabetic nephropathy. It was reported that type 1 diabetic nephropathy patients, compared with type 1 diabetic patients without nephropathy, showed a different DNA methylation pattern at UNC13B [18], an unique gene that was associated with increased risk of diabetic nephropathy [19]. In the meantime, our research showed that high glucose induced hypo-methylation of CTGF gene promoter, leading to higher CTGF protein expression in human glomerular mesangial cells (hMSCs) [20]. We have also demonstrated that CTGF methylation levels were significantly decreased in patients with type 2 diabetic nephropathy, compared with type 2 diabetic patients without nephropathy or healthy control subjects, and that CTGF promoter methylation levels were negatively correlated with CTGF expressions [21].

In the present study, we demonstrated that the increased nuclear export of Dnmt3a is responsible for the hypo-methylation of CTGF in high glucose-stimulated hMSCs. High glucose could activate ERK/MAPK pathway to promote cytoplasmic translocation of Dnmt3a.

## MATERIALS AND METHODS

### Human glomerular mesangial cell culture

hMSC, a classic model cell line widely used in the studies of renal pathophysiology [3], was purchased from China Center for Type Culture Collection (CCTCC), and cultured according to our previously published method [20]. Briefly, the hMSCs were maintained in Dulbecco's modified Eagle's medium supplemented with L-glutamine, 10% heat-inactivated fetal calf serum, 100 units/ml penicillin and 100 μg/ml streptomycin. hMSCs were incubated in a humidified incubator with 5% CO_2_ at 37°C, treated under the conditions of low glucose (5 mM), mannitol (30 mM) or high glucose (30 mM) for 24 h, and subject to further experiments.

### Real-time quantitative PCR for CTGF mRNA expression

hMSCs were collected and total RNAs were extracted using Trizol reagent (Invitrogen). cDNA was synthesized using reverse transcription kit ReverTra Ace qPCR RT Kit (TOYOBO) according to the manufacturer's instruction. Real-time quantitative PCR analysis was performed using the SYBR Green PCR Master Mix (TOYOBO) on an Applied Biosystems 7300 Sequence Detection System. PCR primers were designed using Oligo 6.0 software and synthesized by Shanghai Sangon. The PCR was performed with oligonucleotide primers (R&D System) specifically designed to amplify human CTGF (forward primer: 5′-TTG GCA GGC TGA TTT CTA GG-3′ and reverse primer: 5′-GGT GCA AAC ATG TAA CTT TTG G-3′). Following an initial denaturation at 94°C for 2 min, the cDNA was amplified for 35 cycles with the following setting: denaturation at 94°C for 30 s, annealing at 58°C for 30 s, and elongation at 72°C for 45 s. The amplification ended with a final elongation at 72°C for 10 min. The melting curve was used to confirm the specificity of final amplification products. The relative amounts of CTGF mRNA were expressed as 2^−ΔΔCT^.

### Subcellular protein fractionation

The subcellular protein fractionation was performed according to previously published method [22] with minor modification. Briefly, 500 μl of lysis buffer A (10 mM HEPES, 1.5 mM MgCl_2_, 10 mM KCl, 0.5 mM DTT, 0.05% NP40, pH 7.9) with protease inhibitor cocktail (Sigma) was added to hMSCs cultured on large petri dish. Each dish was scraped thoroughly and the resulting cell lysate was centrifuged at 16000 ***g*** for 10 min at 4°C. The supernatant was saved for analysis of cytoplasmic proteins, and the cell pellet was further re-suspended in 374 μl of Buffer B (5 mM HEPES, 1.5 mM MgCl_2_, 0.2 mM EDTA, 300 mM NaCl, 0.5 mM DTT, 26% glycerol (v/v), pH 7.9) on ice. The suspension was then homogenized on ice with glass homogenizer for 20 times, left on ice for 30 min and centrifuged at 24000 ***g*** for 20 min at 4°C. The supernatant was examined for levels of nuclear proteins using western blot. β-Actin and Lamin B were used as cytoplasmic and nuclear markers respectively.

### Western blotting

hMSCs were treated with control (DMSO), ERK inhibitor VX-11E (0.5 μM, Chemietek), MEK inhibitor PD0325901 (0.5 μM, Sigma) or MEK inhibitor PD98059 (50 μM, Sigma) for 12 h, harvested, and total protein was collected as previously described [20]. Protein concentrations were measured using BCA protein assay kit (Pierce). Total protein, cytoplasmic protein or nuclear protein were separated by SDS/PAGE and were electrotransferred to PVDF membranes. The resulted membranes were blocked with PBS containing 5% milk for 2 h before they were incubated with human anti-CTGF antibody (1:1000, Abcam), anti-Dnmt3a antibody (1:1000, Abcam), anti-MEK antibody (1:1000, Cell Signaling), anti-phosphorylation MEK antibody (1:1000, Cell Signaling), anti-ERK antibody (1:1000, Cell Signaling) or anti-phosphorylation ERK antibody (1:1000, Cell Signaling) at 4°C overnight. β-Actin or Lamin B was used as a loading control. After being washed for three times with TBST, the membranes were further incubated with HRP-conjugated goat anti-mouse or anti-rabbit IgG (1:10000, Santa Cruz) at room temperature for 2 h. Finally, the protein expressions were measured using chemiluminescent staining reagent kits (Supersignal West Femto, Rockford, IL, USA) and the staining images were captured using Image Scanner. Image band intensities were quantified with ImageJ software.

### Immunofluorescence staining

hMSCs were fixed in 4% paraformaldehyde in PBS for 10 min at room temperature and permeabilized with 0.1% Triton X-100 in PBS for another 10 min. After the cells were blocked in bovine serum albumin in PBS for 30 min, they were incubated with human anti-Dnmt3a (1:200, Abcam) antibody at room temperature for 2 h. After PBST wash, cells were incubated with Alexa-conjugated goat anti-rabbit secondary antibody (1:400, Invitrogen) for 1 h at room temperature. Cells were then stained with DAPI for nucleus visualization, with the fluorescent intensity assessed on 10 microscopic fields by digital analysis (Windows MicroImage, version 3.4 CASTI Imaging).

### Chromatin immunoprecipitation

ChIP analysis with hMSCs was performed by ChIP Assay Kit (Upstate Biotechnology) as described earlier [23]. Basically, hMSCs were treated with low glucose (5 mM), mannitol (30 mM) or high glucose (30 mM) for 24 h. After being cross-linked by formaldehyde, cells were washed with PBS, re-suspended in SDS lysis buffer, and briefly sonicated to shear genomic DNA. Immunoprecipitation was performed by adding anti-Dnmt3a antibody (Abcam) to the experimental group or normal mouse IgG to the negative control group. After protein A agarose beads were added, immune complexes were washed and co-precipitated DNA fragments were eluted. Before antibodies were added, a portion of the diluted cell supernatant was taken out as ‘input’ to estimate the amount of DNA in different samples. Recovered DNA was purified by QIAquick PCR purification kit (Qiagen) and the purified DNA was used as PCR template. PCR primers (CTGF) for ChIP assays were as follows: Forward: 5′-GTT GAT GAG GCA GGA AGG TG-3′; Reverse: 5′-CGG TCA TGG TTG GCA CTG-3′. Quantification of Dnmt3a-binding (% input) was performed by determining the amount of specific signal compared with input DNA from three independent experiments. Experimental PCR products of methylation of CTGF promoter were normalized to the PCR products of relevant mannitol treatment.

### 2.7. Data presentation and statistical analysis

Each experiment was repeated for at least three times. All data were presented as mean±S.D. and expressed as fold change over control. Student's *t* test was used for the comparison between two groups. When more than two groups were studied, data were analysed by ANOVA. Differences with *P*<0.05 were considered statistically significant.

## RESULTS

### High-glucose stimulates CTGF expression in hMSCs

**Figure 1:**
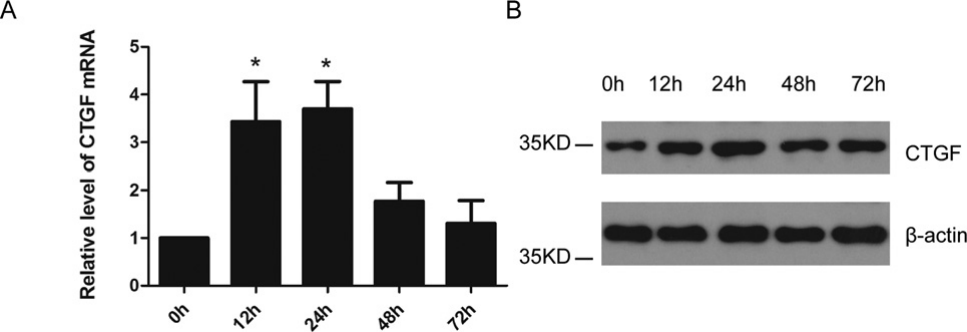
High glucose induces the expression of CTGF in hMSCs (**A**) Real-time quantitative PCR analysis of CTGF mRNA expression level at 0, 12, 24, 48 or 72 h post high glucose treatment. *n*=3. *P<0.05. (**B**) Western blot analysis of CTGF protein expression level at 0, 12, 24, 48 or 72 h post high glucose treatment. *n*=3 and representative image was shown.

A time-based study was carried out to simultaneously examine the mRNA and protein expressions of CTGF in hMSCs cultured with low glucose (5 mM), mannitol (30 mM) or high glucose (30 mM). A significant increase in CTGF mRNA expression was observed at 12 and 24 h post high-glucose stimulus (*P*<0.05) (Figure 1A). In addition, CTGF protein expression level was elevated at 12 h and reached peak value at 24 h post high-glucose stimulus (Figure 1B). However, low glucose or mannitol stimulation did not alter the expression of CTGF at any time point post treatment (0, 12, 24, 48, 72 h) (results not shown).

### High glucose promotes the cytoplasmic translocation of Dnmt3a

**Figure 2:**
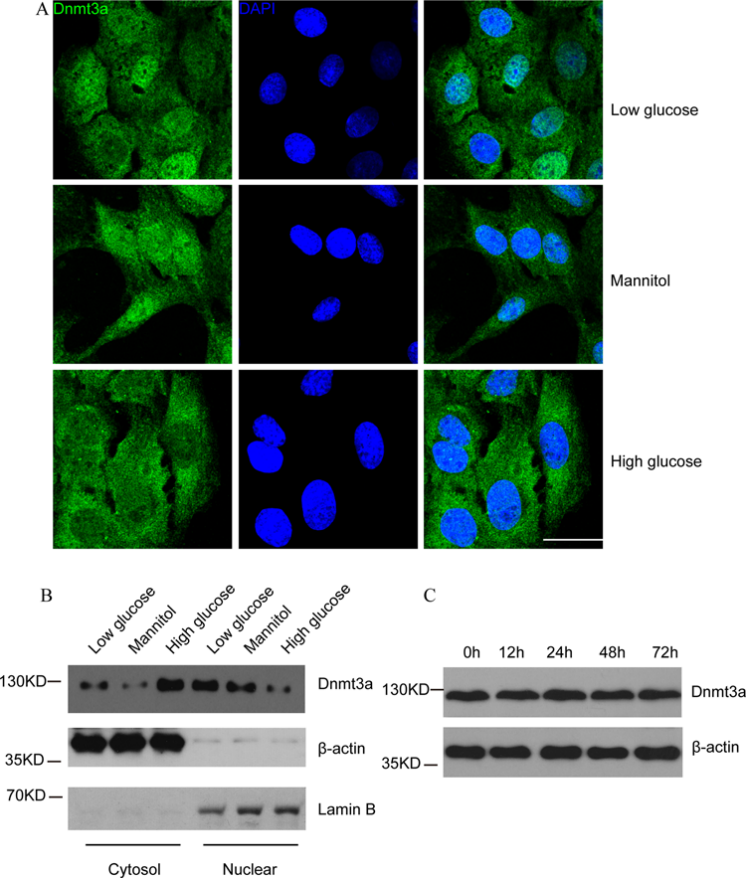
High glucose promotes the cytoplasmic translocation of Dnmt3a in hMSCs (**A**) Immunofluorescence staining of Dnmt3a (green) under low glucose, mannitol or high glucose conditions for 24 h were shown. DAPI (blue) was used to stain the nucleus. At least 10 microscopic fields were assessed in each experiment. Images are representative of three independent experiments. Scale bar, 10 μm. (**B**) Western blot analysis of cytoplasmic and nuclear Dnmt3a protein levels at 24 h post high glucose treatment. β-Actin was used as the internal control for cytoplasmic portion and Lamin B was used as the internal control of nuclear portion. *n*=3 and representative image was shown. (**C**) Western blot analysis of total Dnmt3a protein levels at 0, 12, 24, 48 or 72 h post high glucose treatment. *n*=3 and representative image was shown.

Our previous research revealed that high glucose induced hypo-methylation of CTGF gene promoter both *in vitro* [20] and *in vivo* [21]. It is possible that high glucose affects the expression or function of Dnmts, which in turn changes CTGF methylation status. There are three major Dnmts in mammalian cells, Dnmt1, which acts as a maintenance methyltransferase, as well as Dnmt3a and Dnmt3b, which mediate *de novo* methylation [24]. To find out the specific Dnmt that responds to high-glucose stimulation, a time-dependent induction experiment was performed. Neither Dnmt1 (Supplementary Figures S1A and S1B) nor Dnmt3b (Supplementary Figures S1A and S1C) showed different expression patterns at 12, 24, 48, or 72 h post induction, as compared with their initial expression profiles. Interestingly, an increased cytosolic translocation of Dnmt3a was observed after high glucose treatment, as compared with low glucose or mannitol treatment (Figure 2A). This result was further confirmed by western blotting analysis. A concomitant increase in cytoplasmic Dnmt3a and decrease in nuclear Dnmt3a levels were observed after high glucose treatment (Figure 2B). However, compared with 0 h, total Dnmt3a expression did not change at 12, 24, 48 or 72 h post high-glucose stimulus (Figure 2C). These results suggested that high glucose promoted cytoplasmic translocation of Dnmt3a, which might lead to the hypo-methylation [20] and the increased expression of CTGF (Figures 1A and 1B).

### High glucose reduces the binding between Dnmt3a and CpG island of CTGF

**Figure 3:**
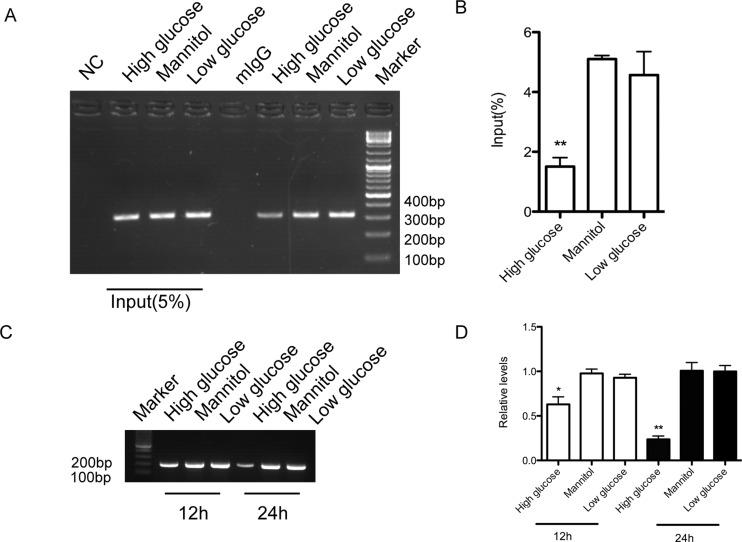
High glucose decreases the binding of Dnmt3a to the CpG island of CTGF promoter (**A**) ChIP assay with anti-Dnmt3a antibody performed 24 hours after high glucose, mannitol, or low glucose treatment. *n*=3 and representative image was shown. (**B**) Quantification of ChIP assay based on three independent experiments. **P<0.01. (**C**) Methylation specific primer was use to amplify methylated CpG island of CTGF promoter at 12 and 24 h after high glucose, low glucose or mannitol treatment. *n*=3 and representative image was shown. (**D**) Quantification of methylation of CpG island of CTGF promoter based on 3 independent experiments. **P<0.01.

Since high glucose induces both hypo-methylation of CTGF and cytoplasmic translocation of Dnmt3a, it is important to understand whether Dnmt3a indeed binds to the CpG island of CTGF promoter and whether this binding is a direct result of high glucose induction. To answer the above questions, bioinformatics analyses were carried out to search for putative CpG island on CTGF promoter. As we extended the analysed sequence of CTGF gene to 5’-UTR region, a GC-rich region that apparently matched the characteristics of putative CpG island was identified. This region extends from -97 to +151 around the transcription start site (+1), giving rise to a 249-bp product. ChIP assay was applied in this experiment. As shown in Figure 3(A), with low glucose, Dnmt3a was clearly bound to the putative CpG island on CTGF gene promoter. Nevertheless, high glucose significantly lowered the binding between Dnmt3a and the CpG island of CTGF (Figures 3A and 3B). Specific primer for methylated DNA was employed to detect methylation of CpG island of CTGF promoter. The results showed that the methylation of CpG island of CTGF promoter was reduced in a time-dependent manner in the presence of high glucose, as compared with hMSCs treated with low glucose or mannitol (Figures 3C and 3D).

### ERK/MAPK signalling pathway contributes to high glucose-induced cytoplasmic translocation of Dnmt3a

**Figure 4:**
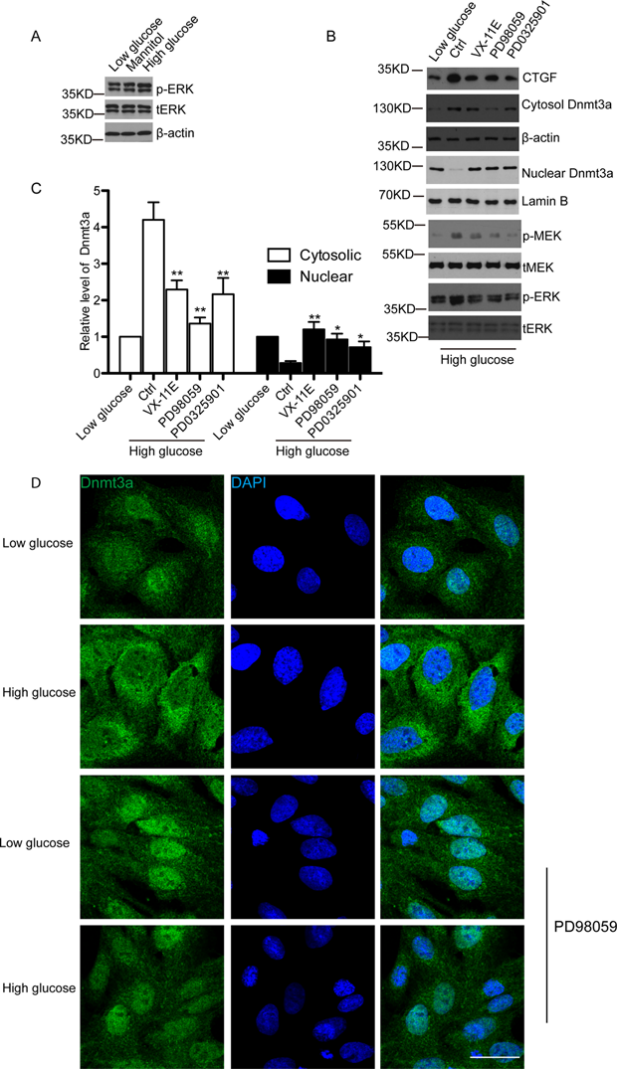
ERK/MEK signalling pathway contributes to cytoplasmic translocation of Dnmt3a in high glucose-treated hMSCs (**A**) Western blot detected the pERK and total ERK (tERK) in low glucose, mannitol or high glucose-treated hMSCs. β-Actin was used as a loading control. *n*=3 and representative image was shown. (**B**) Western blot analysis of CTGF, pMEK, total MEK (tMEK), pERK, tERK, cytoplasmic and nuclear Dnmt3a protein levels at 24 h post high glucose treatment followed by 12 h treatment of control, 0.5 μM ERK inhibitor VX-11E, 50 μM MEK inhibitor PD98059 or 0.5 μM MEK inhibitor PD0325901. β-Actin was used as the internal control for cytoplasmic portion and Lamin B was used as the internal control of nuclear portion. *n*=3 and representative image was shown. (**C**) Quantitative analysis of (B) by ImageJ software based on three independent experiments. *P<0.05. **P<0.01. (**D**) Immunofluorescence staining of Dnmt3a (green) under low glucose, mannitol or high glucose conditions for 24 h with or without 50 uM PD98059. DAPI (blue) was used to stain the nucleus. At least 10 microscopic fields were assessed in each experiment. Images are representative of three independent experiments. Scale bar, 10 μm.

To further elucidate possible signalling pathway regulating translocation of Dnmt3a, we evaluated the expression of phosphorylated ERK (pERK) and phosphorylated MEK (pMEK). We observed that pERK and pMEK were increased in hMSCs in high glucose medium comparing to that in low glucose or mannitol medium (Figures 4A and 4B). Next, we investigated whether MEK inhibitors could reverse the increased cytoplasmic translocation of Dnmt3a upon high glucose treatment. PD98059, a highly selective inhibitor of MEK1 activation and the MAP kinase cascade [25], was used to treat the hMSCs. Our results demonstrated that increased cytoplasmic Dnmt3a and CTGF induced by high glucose was reversed when pERK was inhibited by PD98059 (Figures 4B and 4C). Consistently, similar results were observed with an ERK inhibitor, VX-11E [26], and a second MEK inhibitor, PD0325901 [27] (Figures 4B and 4C). Additionally, immunofluorescence staining of hMSCs also indicated that cytoplasmic translocation of Dnmt3a induced by high glucose was blocked by PD98059 (Figure 4D).

## DISCUSSION

Using hMSC, a model cell type that is crucial in the development of glomerular fibrosis, which contributes to the pathogenesis of diabetic nephropathy [28], we found that high glucose induced the expressions of both CTGF mRNA (Figure 1A) and protein (Figure 1B), which is consistent with previous observation from human renal fibroblast cell [29]. Emerging evidence has indicated that CTGF is involved in the development of glomerulosclerosis, tubuloepithelial fibrosis and tubular atrophy, diseases that are characterized by the accumulation of extracellular matrix proteins, such as fibronectin and collagens. Specifically, CTGF has been shown to induce the expressions of fibronectin and type IV collagen [30]. In addition, inhibition of CTGF could lower the production of fibronectin and the severity of renal fibrosis [31]. Therefore, understanding the mechanisms underlying high glucose-induced CTGF up-regulation would not only deepen our knowledge of diabetic nephropathy, but also provide insight into novel strategies to prevent diabetic nephropathy progression.

Epigenetic mechanisms play an important role in the development of diabetic nephropathy [32]. Epigenetic inheritance system is essential for the metabolic memory, which refers to the phenomenon that diabetes complications persist despite returning to normal glucose [33]. Clinical evidence has shown that transient exposure to hyperglycaemia might induce possible epigenetic changes, which could lead to the hyperglycaemic memory and result in persistent risk for diabetic nephropathy [34]. It has been reported that changes in DNA methylation patterns were highly correlated with the onset of diabetic nephropathy [18]. Interesting, we have previously discovered that the methylation level of CTGF gene promoter was greatly reduced in high glucose-treated hMSCs [20] and in diabetic patients with nephropathy [21]. However, little is known about the regulatory mechanism of CTGF gene hypo-methylation under hyperglycaemia. In mammals, DNA methylation, which includes the addition of methyl group to adenine and cytosine, is predominantly controlled by Dnmts, including the maintenance methyltransferase Dnmt1, and the *de novo* DNA methyltransferases Dnmt3a and Dnmt3b [35]. Additionally, Dnmt1, Dnmt3a and Dnmt3b are coordinately expressed in adult human kidney tissues [36]. In the present study, we found that the total expression of Dnmt1, Dnmt3a or Dnmt3b (Supplementary Figures S1A and S2C) remained unchanged after high glucose induction. However, a significantly increased cytoplasmic translocation of Dnmt3a, but not Dnmt1 or Dnmt3b, was shown under hyperglycaemia (Supplementary Figure S1B, Figures 2B and 2C), suggesting that the nuclear import of Dnmt3a, which is required for Dnmt3a to methylate DNA, is the key factor that control high glucose-induced reduction in CTGF methylation.

In mammals, cytosine methylation takes place at cytosines located at CpG island, which is a short 0.5–4 kb sequence with rich GC content (40–60%) [37]. Our study revealed that the CTGF gene promoter contains a dense CpG island, which extends from -97 to +151 around the transcription start site (+1). This is consistent with recent findings in acute lymphoblastic leukaemia cell lines [38]. Moreover, it has been shown that the methylation profile around the CpG island of CTGF correlated inversely with the expression of CTGF gene [39]. Although Dnmt3a and Dnmt3b have similar *in vitro* DNA methylation properties and overlapping functions, our data clearly demonstrated that Dnmt3a (Figure 3), but not Dnmt3b (results not shown), has the capacity to bind to this CpG island and contributes to the methylation of CTGF promoter. To our best knowledge, this is the first report to clarify the specific Dnmt subtype that is responsible for the methylation of CTGF gene, although it remains unclear what factors determine the specificity of Dnmts activities.

Several mechanisms could modulate the expression, subcellular translocation or the functions of Dnmts. For example, Dnmt1, Dnmt3a and Dnmt3b all contain nuclear localization signals (NLS) in their N-terminus, which are important for the nuclear translocation of Dnmts [40]. Deacetylation of lysines located in the NLS of Dnmt1 changes the methyltransferase activities of Dnmt1 [41], and the nuclear translocation of Dnmt1 is increased through AKT-mediated phosphorylation of Dnmt1 NLS [42]. Interestingly, studies have shown that the inhibition of ERK/MAPK pathway could decrease the expression of Dnmt1 [43] or Dnmt3b [44]. Furthermore, MEK inhibitor PD985059 greatly affects the nuclear and cytoplasmic distribution of Dnmt3a in smooth muscle cells [45]. On the other hand, it is well known that ERK/MAPK signalling pathway closely correlates with glomerular and tubulointerstitial lesions and contributes to diabetic nephropathy [46,47]. Inhibition of ERK/MAPK pathway markedly attenuates renal injury and diabetic nephropathy [48,49]. Therefore, ERK/MAPK pathway might directly contribute to the observed cytoplasmic translocation of Dnmt3a and increased CTGF expression induced by high glucose in hMSCs. Indeed, we demonstrated in the present study that inhibition of ERK/MAPK pathway via three distinct inhibitors, including VX-11E, PD98059 and PD0325901, significantly increased the nuclear translocation of Dnmt3a in hMSCs, and consequently inhibited CTGF up-regulation induced by high glucose (Figure 4). This result is consistent with previous studies showing that ERK/MAPK pathway mediates high glucose-induced CTGF up-regulation [50] or increased CTGF excretion [51] in HK-2 cells. Although it remains to be further explored whether ERK/MEK signalling regulates the subcellular translocation of Dnmt3a post-transcriptionally or through protein-protein interactions, our study clearly reveals a novel regulatory mechanism of CTGF expression in diabetic nephropathy and strongly supports the therapeutic value of ERK/MAPK inhibitors in the prevention and treatment of diabetic nephropathy.

In summary, high-glucose stimulation activates ERK/MEK pathway and subsequently increases the cytoplasmic translocation of Dnmt3a, which contributes to the hypo-methylation of CTGF promoter and the increased expression of CTGF in diabetic nephropathy.

## Abbreviations

CTGF: connective tissue growth factor
DN: diabetic nephropathy
Dnmt: DNA methyltransferase
ESRD: end-stage renal disease
hMSC: Human glomerular mesangial cell
pERK: phosphorylated ERK
pMEK: phosphorylated MEK
tERK: total ERK
